# Allergen immunotherapy

**DOI:** 10.1186/s13223-024-00935-2

**Published:** 2024-12-16

**Authors:** Jean-Nicolas Boursiquot, Rémi Gagnon, Jaclyn Quirt, Anne K. Ellis

**Affiliations:** 1 Division of Allergy and Clinical Immunology, Department of Medicine, Centre Hospitalier Universitaire de Québec, Université Laval, Québec, Québec Canada; 2 Division of Clinical Immunology & Allergy, Department of Medicine, McMaster University, Hamilton, ON Canada; 3 Division of Allergy & Immunology, Department of Medicine, Queen’s University, Kingston, ON Canada

## Abstract

Allergen immunotherapy (AIT) is a potentially disease-modifying therapy that is effective for the treatment of allergic rhinitis/conjunctivitis, allergic asthma and stinging insect hypersensitivity. The decision to proceed with AIT should be made on a case-by-case basis, based on a comprehensive evaluation of the patient, allergy testing and a thorough discussion with the patient about treatment goals, risks vs. benefits, and long-term commitment to the treatment plan. For those with allergic rhinitis and/or asthma, it is also important to consider individual patient factors, such as the degree to which symptoms can be reduced by avoidance measures and pharmacological therapy, the amount and type of medication required to control symptoms, the adverse effects of pharmacological treatment, and patient preferences.

Since AIT is associated with a risk of anaphylaxis, it should only be prescribed by physicians who are adequately trained in the treatment of allergic conditions. Furthermore, for subcutaneous therapy, injections must be given under medical supervision in clinics that are equipped to manage anaphylaxis. In this article, we review the indications and contraindications, patient selection criteria, and details regarding the administration, safety and efficacy of AIT for allergens other than foods. Immunotherapy for food allergy will be discussed in the *Oral Immunotherapy* article in this supplement.

## Key take-home messages

- AIT is a potentially disease-modifying therapy that is effective for the treatment of allergic rhinitis/conjunctivitis, allergic asthma and stinging insect hypersensitivity, as well as atopic dermatitis associated with HDM or aeroallergen sensitivity.
- AIT is contraindicated in patients with uncontrolled or severe asthma, or those with significant co-morbid cardiovascular disease.
- The use of beta-blockers is generally contraindicated for environmental AIT and is a relative contraindication for venom immunotherapy. Consideration of risk-benefit should be taken for concomitant use of ACE inhibitors.
- The decision to proceed with AIT should be made on a case-by-case basis based on a thorough clinical evaluation, allergy testing and patient discussions regarding risks vs. benefits and long-term commitment to the treatment plan. In patients with allergic rhinitis and/or asthma, it is also important to take into account individual patient factors such as disease severity, efficacy of avoidance measures and pharmacological therapy, and patient preferences for disease-modifying therapy vs. conventional treatment.
- AIT carries the risk of anaphylactic reactions and, therefore, should only be prescribed by physicians who are adequately trained in the treatment of allergy.
- SCIT must be given under medical supervision in clinics that are equipped to manage life-threatening anaphylaxis.
- SLIT is available in Canada for the treatment of HDM, grass, birch and ragweed allergy. It offers multiple potential benefits over SCIT including the comfort of avoiding injections, the convenience of home administration, and a favourable safety profile.

## Introduction

Allergen immunotherapy (AIT) is a well-established and effective treatment for common allergic conditions, particularly allergic rhinitis/conjunctivitis, allergic asthma and stinging insect hypersensitivity [1–9]. Note that this article focuses on AIT for allergens other than foods. For a review of immunotherapy for food allergy, please refer to the *Oral Immunotherapy* article in this supplement.

There are two different routes of administration available for AIT in Canada: subcutaneous (SCIT) and sublingual (SLIT). SCIT typically involves the administration of gradually increasing quantities of the patient’s relevant allergen(s) until a dose is reached that is effective in inducing immunologic tolerance to the allergen(s). SCIT should be prescribed and monitored by an experienced allergist, with all injections given under physician supervision [6]. SLIT involves regular self-administration (under the tongue) of allergen extract at home and, unlike SCIT, does not require extensive ‘up-dosing’. SLIT should only be prescribed and initiated by physicians with adequate training and experience in the treatment of respiratory allergic diseases.

The primary objectives of AIT are to decrease the symptoms triggered by allergens and to prevent recurrence of the disease in the long-term. Currently, it is the only identified disease-modifying intervention for allergic disease [5–7]. This article will review the mechanisms of AIT, its indications and contraindications, patient selection criteria, and the administration, safety and efficacy of this form of therapy.

## Mechanisms of immunotherapy

Immunologic changes that occur during AIT are complex and not completely understood. Successful immunotherapy has been associated with a shift from T helper cell type-2 (Th2) immune responses, which are associated with the development of atopic conditions, to a better balance with more Th1 immune responses [5, 8, 9]. It is also associated with the production of T regulatory cells that produce the anti-inflammatory cytokine, interleukin 10 (IL-10), amongst others such as transforming growth factor (TGF)-beta. IL-10 has been shown to reduce levels of allergen-specific immunoglobulin E (IgE) antibodies, increase levels of immunoglobulin G4 (IgG4) (“blocking”) antibodies that play a role in secondary immune responses, and reduce the release of pro-inflammatory cytokines from mast cells, eosinophils and T cells. AIT has also been found to decrease the recruitment of mast cells, basophils, and eosinophils to the skin, nose, eye, and bronchial mucosa after exposure to allergens, and reduce the release of mediators, such as histamine, from basophils and mast cells [5, 8, 9].

There are differences in mechanisms between SCIT and SLIT. In SCIT, the IgE-inhibitory activity is predominantly mediated by IgG4, whereas in SLIT, local nasal and systemic blocking antibodies are largely related to IgA [8]. Research surrounding the mechanisms of SCIT and SLIT is still ongoing and will help further elucidate how these therapies exert their beneficial effects in allergic diseases.

## Indications

AIT is indicated in patients with allergic rhinitis/conjunctivitis and/or allergic asthma who have evidence of specific IgE antibodies to clinically relevant allergens, including in those who experience undesirable side effects of pharmacotherapy, who do not want ongoing/long-term pharmacotherapy and/or who wish to receive a potentially disease-modifying treatment (see Table 1) [6]. AIT may also be effective in select patients with atopic dermatitis that is associated with house dust mite (HDM) or aeroallergen sensitization [6]. Skin prick testing (SPT) is the preferred method of testing for specific IgE antibodies. In-vitro measurement of allergen-specific IgE testing is a reasonable alternative to SPT, however, SPTs are generally considered to be more sensitive and cost effective than serum-specific IgE tests [5, 9].

**Table 1:** Indications, available allergens, contraindications and special considerations for AIT [6]

	SCIT	SLIT	Venom immunotherapy
Indications	• Patients with symptoms of rhinitis, conjunctivitis and/or asthma induced by allergen exposure; includes patients who: — Do not want ongoing or long-term pharmacotherapy — Experience undesirable side effects with pharmacotherapy — Wish to receive a potentially disease-modifying therapy • Patients with atopic dermatitis associated with HDM or aeroallergen sensitization (may be considered)		• Individuals of all ages with anaphylactic reactions to stinging insects • Individuals with troublesome, likely recurrent, large local reactions
Available allergens	• Birch • Grass • Ragweed • HDM • Cat • Dog • *Alternaria* • Cockroach	• Birch • Grass • Ragweed • HDM *(see* Table 2* for approved products and age indications)*	• Honeybee • Yellow jacket • Hornet • Wasp • Fire ants
Contraindications	• Poorly controlled or severe asthma • Significant co-morbid diseases such as cardiovascular disability • Caution advised and shared decision making required for the concomitant use of beta-blockers (generally considered a contraindication to environmental AIT, and a relative contraindication to venom immunotherapy) • Eosinophilic esophagitis (SLIT) • Inflammation, injury or surgical intervention in oral cavity (SLIT) • Previous severe, systemic reaction to AIT		
Special populations	Immunotherapy may be used in the following special populations, but there may be specific clinical issues that need to be addressed in these groups: • Children < 5 years of age • Pregnant patients • Elderly patients • Patients with malignancy, immunodeficiency or autoimmune diseases		

AIT: allergen immunotherapy; HDM: house dust mites; SCIT: subcutaneous immunotherapy; SLIT: sublingual immunotherapy

**Table 2:** SLIT products approved in Canada [6, 13–17]

Product	Allergen	Age indication (year)*	Dose	Administration
Oralair^®^	grass	5–50	First: 100 IR Second: 200 IR Subsequent: 300 IR	• Take initial dose of 100 IR in office, 16 weeks before onset of grass pollen season; • Take second dose of 200 IR at home the following day; • Then, take 300 IR per day at home until end of grass pollen season.
Grastek^®^	grass	5–65	2800 BAU	• Take initial dose in office, 8–12 weeks before grass pollen season; • Then, take daily dose at home until end of grass pollen season.
Ragwitek^®^	ragweed	5–65	12 amb a 1-U	• Take initial dose in office, 12 weeks before ragweed season; • Then, take daily dose at home until end of ragweed season.
Acarizax™	dust mite	12–65	12 SQ-HDM^†^	• Take initial dose in office, anytime throughout the year; • Then, take daily dose at home for 3–5 years if effective.
Itulatek^®^	birch	18–65	12 SQ-Bet^‡^	• Take initial dose in office, 16 weeks before birch pollen season; • Then, take daily dose at home throughout season.

BAU: Bioequivalent Allergy Units; IR: index of reactivity; U: units

* Please note that the age indications reflect the groups studied in clinical trials. Often SLIT is indicated outside of these age indications

†SQ-HDM is the dose unit for Acarizax™. SQ is a method for standardization on biological potency, major allergen content and complexity of the allergen extract. HDM is an abbreviation for house dust mite

^‡^SQ-Bet is the dose unit for Itulatek^®^. SQ is a method for standardization on biological potency, major allergen content and complexity of the allergen extract. Bet is an abbreviation for *Betula verrucosa* or birch

Venom immunotherapy is indicated in individuals of all ages with anaphylactic reactions to stinging insects, as well as in individuals with troublesome, likely recurrent, large local reactions [6, 10] (Table 1). In addition to assessing for venom-specific IgE, consideration should also be given to measuring basal serum tryptase in patients who are candidates for venom immunotherapy since an elevated level of this serine proteinase has been shown to be an important risk factor for severe reactions before, during, and after immunotherapy [10]. Lifelong venom immunotherapy may be appropriate in certain situations, such as in those with elevated serum basal tryptase levels and/or a history of severe systemic reactions to stinging insects [6, 11]. Severe systemic reactions to Hymenoptera (the classification of insects that includes bees and wasps) venom are relatively uncommon, but can be fatal. The purpose of venom immunotherapy is to reduce the severity of the reactions and the risk of fatality, and to improve patient quality of life by allowing the patient to work or play outdoors without being concerned about the possibility of experiencing a serious allergic reaction [5, 10].

## Contraindications

In general, all forms of AIT are contraindicated in patients with medical conditions that increase the patient’s risk of dying from treatment-related systemic reactions, such as those with severe or poorly controlled asthma or significant cardiovascular diseases (e.g., unstable angina, recent myocardial infarction, significant arrhythmia, and uncontrolled hypertension) [6, 9] (see Table 1). AIT is also contraindicated in those with a previous severe systemic reaction to immunotherapy.

Exposure to beta-blockers has been associated with more serious and treatment-resistant anaphylaxis [9, 10]. Therefore, the use of beta-blockers is generally a contraindication to environmental AIT, and a relative contraindication to venom immunotherapy [6, 12]. In patients with life-threatening stinging insect hypersensitivity, venom immunotherapy may be considered even in those using beta-blockers because the fatal risk associated with an insect sting is greater than the risk of an immunotherapy-related systemic reaction. Additionally, angiotensin-converting enzyme (ACE) inhibitors have been associated with a greater risk for more severe reactions from venom immunotherapy as well as stings [9, 10], although this finding is not consistent. Therefore, consideration should be given to discontinuing ACE inhibitors in patients undergoing venom or inhalant immunotherapy.

In addition to the above-mentioned contraindications, SLIT is also contraindicated in patients who have active inflammatory conditions in the oral cavity (e.g., oral lichen planus with ulcerations, severe oral candidiasis, dental extraction) [13–17]. SLIT has been also associated with new or worsening eosinophilic esophagitis (EoE; see *Eosinophilic Esophagitis* article in this supplement) [6, 18]. Pre-existing EoE or a history of this disorder is a contraindication to the use of SLIT.

## Special considerations

Although there is no specific upper or lower age limit for initiating AIT, special consideration should be given to its use in children under 5 years of age and the elderly [6, 9]. AIT is effective in children and is often well tolerated. In fact, grass and ragweed SLIT are indicated for children as young as 5 years old. However, young children may have difficulty cooperating with the immunotherapy regimen and injections. Therefore, physicians need to weigh the risks and benefits of therapy in this patient population. The risks vs. benefits of immunotherapy also need to be considered in the elderly since these patients often have comorbid medical conditions, such as cardiovascular disease, that may increase the risk of experiencing immunotherapy-associated adverse events.

Special consideration should also be given to the use of AIT in pregnant women, and in patients with malignancy, or immunodeficiency/autoimmune diseases (see Table 1) [6]. Immunotherapy is generally not initiated in pregnant women; however, it can be continued in women who have been on treatment prior to becoming pregnant, after discussion about the risks of anaphylaxis in pregnancy [9, 19]. Finally, some physicians may feel hesitant about modifying the immune system in patients with autoimmune disorders, immunodeficiency syndromes, or malignant disease. However, there is no convincing evidence that AIT poses any harm to these patients, provided that the risks and benefits of therapy have been considered [9].

## Efficacy

### Allergic rhinitis

Both SCIT and SLIT are effective for the treatment of allergic rhinitis/conjunctivitis caused by HDM, birch, grass and ragweed pollens [3, 5, 6, 20–23]. SCIT has also been shown to be effective for the treatment of allergic rhinitis caused by Alternaria, cockroach, and cat and dog dander [3, 5, 6, 24, 25]. Patients’ symptoms often improve even when they were resistant to conventional drug therapy.

Evidence suggests that at least 3 years of AIT (SCIT or SLIT) provides beneficial effects in patients with allergic rhinitis that can persist for several years after discontinuation of therapy [23, 26–30]. In Canada, most allergists consider stopping immunotherapy after 5 years of adequate treatment. Recent data has made it clear that only 2 years of immunotherapy, either via the subcutaneous or sublingual route, is not sufficient to provide long-lasting effects [30, 31].

While there are few studies directly comparing the efficacy of SCIT vs. SLIT, a 2013 meta-analysis that indirectly compared systematic reviews found that both forms of immunotherapy had significant benefits over placebo, however one modality could not conclusively be deemed superior to the other [32]. A more recent network meta-analysis of 26 randomized clinical trials also found both forms of immunotherapy to be effective in improving clinical symptoms and reducing medication use in patients with allergic rhinitis, although SCIT was found to be somewhat more effective than SLIT in controlling allergic rhinitis symptoms [33].

Children with allergic rhinitis are at increased risk of developing asthma later on in life when compared to those without allergic rhinitis [34]. Evidence suggests that AIT may reduce the risk for the future development of asthma in these children [5].

### Asthma

SCIT has been shown to be effective against allergic asthma caused by grass, ragweed, HDM, cat and dog dander, and Alternaria [6, 35]. A Cochrane review of 88 randomized controlled trials examining the use of SCIT in asthma management confirmed its efficacy in reducing asthma symptoms and the use of asthma medications, and improving airway hyperresponsiveness [1]. Similar benefits on asthma outcomes have been noted with SLIT [36, 37], which is available for HDM, grass, birch and ragweed allergies. In large-scale clinical trials of HDM-SLIT in patients with asthma and HDM allergic rhinitis, SLIT was associated with a reduction in ICS use, decreased requirements for ICS, and increased time to exacerbation during ICS reduction in those with suboptimally controlled asthma [38, 39]. Based on these findings, current Global Initiative for Asthma (GINA) guidelines recommend that HDM-SLIT be considered in adult HDM-sensitized patients with allergic rhinitis with persistent asthma symptoms provided that forced expiratory volume in 1 s (FEV_1_) is more than 70% predicted [40]. Ragweed and grass SLIT have also been shown to improve asthma symptom scores and reduce medication use in pediatric patients with allergic asthma [41, 42].

Evidence suggests that AIT may also prevent the onset of asthma in atopic individuals [43, 44]. One study in children with grass and/or birch pollen allergy found that only 26% of subjects treated with immunotherapy developed asthma 3 years after completion of treatment compared to 45% who were not treated with immunotherapy [44]. AIT may also modify the progression of established asthma in children. A study published in the 1960s found that 70% of treated children no longer had asthma 4 years after completing immunotherapy compared to 19% of untreated control subjects, and these results were sustained up to 16 years of age [45]. However, there is no current evidence that immunotherapy influences the evolution of established asthma in adults.

### Atopic dermatitis

There is evidence indicating that immunotherapy can be effective for atopic dermatitis when this condition is associated with HDM or aeroallergen sensitivity [6, 9, 46, 47]. A recent systematic review and meta-analysis of 23 trials including 1957 adult and pediatric patients sensitized to HDM and aeroallergens found both SCIT and SLIT to improve AD severity and patient quality of life [47].

### Venom immunotherapy

Venom immunotherapy provides rapid protection against Hymenoptera stings, and greatly reduces the risk of systemic reactions in stinging insect-sensitive patients, with an efficacy of up to 98% [10]. There is a residual risk of systemic reactions of approximately 5% after completion of venom immunotherapy; however, when reactions to stings do occur following completion of therapy, they are typically mild [10]. Clinical features that have been associated with a greater likelihood of relapse following the discontinuation of venom immunotherapy include: very severe reactions to a sting, systemic reactions during immunotherapy (to injections or stings), elevated basal serum tryptase levels, frequent unavoidable exposure, severe honeybee allergy, and treatment duration of less than 5 years [10].

## Patient selection

The decision to proceed with AIT should be made on a case-by-case basis based on clinical evaluation and physical examination of the patient, appropriate in vivo and in vitro testing to identify specific sensitivity to relevant allergen(s), and a detailed discussion with the patient about treatment goals and long-term commitment to the treatment plan. For those with allergic rhinitis and/or asthma, it is also important to take into account individual patient factors, such as the degree to which symptoms can be reduced by avoidance measures and pharmacological therapy, the amount and type of medication required to control symptoms, and the adverse effects of pharmacological treatment [9]. Some patients may prefer immunotherapy to a pharmacologic treatment approach, and unsuccessful treatment with pharmacotherapy is not a requirement to initiate immunotherapy [6].

Patients selected for immunotherapy should be cooperative and adherent. Those who have a history of nonadherence or who are mentally or physically unable to communicate clearly with the treating physician may be poor candidates for immunotherapy. Inability to communicate effectively with the physician will make it difficult for the patient to report signs and symptoms suggestive of systemic reactions [9].

### Venom hypersensitivity

Before deciding to proceed with venom immunotherapy, it is important to consider the natural history of venom allergy. Patients who have experienced systemic symptoms after a sting are at much greater risk of severe systemic reactions on subsequent stings compared with patients who have had only local reactions. The frequency of systemic reactions to stings ranges between 4 and 10% in those with a history of large local reactions compared to 25–75% in those who have had a previous systemic reaction [10]. In general, children are at lower risk of repeated systemic reactions, as are those with a history of milder reactions [10].

It is also important to consider occupational and geographic factors that may increase the likelihood of future stings. For example, bee stings are much more common in beekeepers, their families, and their neighbours. Yellow-jacket stings are more common in certain occupations such as bakers, grocers and outdoor workers [5].

### Allergic rhinitis

According to current Canadian clinical practice recommendations for allergic rhinitis, AIT is an option for patients who do not achieve symptom control with pharmacotherapy or who prefer this form of therapy [48] (see *Allergic Rhinitis* article in this supplement). It can be introduced at any point in the treatment journey and does not need to be reserved for those with the most severe disease [48]. Patients with allergic rhinitis who are unable to sleep because of symptoms or whose symptoms interfere with their work or school performance despite the use of pharmacotherapy and allergen avoidance measures are particularly good candidates for immunotherapy. Those that experience adverse side effects from pharmacological therapy, such as nosebleeds from intranasal steroids or excessive drowsiness from antihistamines, and those who find pharmacotherapy inconvenient or ineffective and/or who prefer a disease-modifying approach over pharmacotherapy, may also be appropriate candidates for AIT [3, 5]. A flow diagram for the management of allergic rhinitis is provided in the *Allergic Rhinitis* article in this supplement.

### Asthma

According to current GINA guidelines, AIT may be considered for all patients with asthma who have clinically significant sensitization to aeroallergens [40]. However, to reduce the risk of serious reactions, asthma symptoms must be controlled and FEV_1_ should be greater than 70% predicted at the time immunotherapy is administered. Please refer to the *Asthma* article in this supplement for asthma management algorithms.

## Immunotherapy administration and schedules

AIT carries the risk of anaphylactic reactions and, therefore, should only be prescribed by physicians who are adequately trained in the treatment of allergy and the use of immunotherapy (such as allergists and immunologists). SCIT must be given where a physician is present, and in clinics that are equipped to manage possible life-threatening reactions. For SLIT, the first dose of therapy is given under physician supervision. The prescribing physician may choose to provide the patient with an epinephrine autoinjector, though this is not generally required.

Before immunotherapy is started, patients should understand its nature, benefits, risks, and costs. Counseling should also include the expected onset of efficacy and duration of treatment, as well as the risk of anaphylaxis and importance of adhering to the immunotherapy schedule. An assessment of the patient’s current health status should be made before the administration of immunotherapy to determine whether there have been any recent changes in the patient’s health that may require modifying or withholding treatment (e.g., uncontrolled/symptomatic asthma or exacerbation of allergy symptoms) [9].

### Subcutaneous immunotherapy (SCIT)

Allergen extracts are commercially available for most of the commonly recognized allergens (e.g., grass and tree pollen, house dust mites, insect venom). When possible, standardized extracts should be utilized to prepare treatment sets since the efficacy and safety of immunotherapy are dependent on the quality of the allergen extracts used [6]. In choosing the components for a clinically relevant allergen immunotherapy extract, the physician must also be familiar with local and regional aerobiology and indoor and outdoor allergens, paying special attention to potential allergens in the patient’s own environment. Table 3 provides the timing of common pollen and mould allergens across the various geographic regions in Canada [6]. It is important to note that peak pollen seasons are continually evolving due to climate and environmental changes which will necessitate ongoing updates to the geographic and temporal patterns listed in the table.

**Table 3:** Timing and concentration of suspect pollens and mould spores in various geographic areas across Canada [6]

	Tree pollens	Grass pollens	Weed pollens	Mould spores
British Columbia (Coastal)	• Season: early February to mid-July • Primarily alder, birch and poplar • Others such as elm and oak may also contribute	• Season: end of April/ beginning of May to September • Highest grass concentrations: early June to mid-July	• Not generally significant • No native ragweed	• Present throughout the year except for few weeks when ground remains frozen all day • Increase in September and October • Most prevalent spores: Cladosporium and Basidiomycetes
British Columbia (Interior)	• Season: late March to mid-July • Primarily willow, birch, and poplar	• May start in early May in Southern BC • Occurs up to a month later in Northern BC	• Sagebrush can occur in September in Southern BC • Ragweed is minimal	• Cladosporium can occur from April to late fall
Prairie Provinces	• Season: first week of April until June • Primarily birch and poplar • Alder, maple, elm, oak, ash and willow may contribute	• Season: mid-May to end of September • Highest concentration: usually mid-June to early July	• Primarily nettles and sage brush • Some ragweed, especially in Manitoba	• Can occur through spring, summer, and early fall (Alternaria, Cladosporium)
Ontario and Quebec	• Season starts early April in southern Ontario and Quebec; may occur 4–6 weeks later in northern parts • In Ontario, primarily: mulberry, maple, box elder, poplar, willow, oak, beech, birch, alder, and ash; walnut, hickory, elm sycamore, pine and juniper may also contribute • In Quebec, primarily: ash, poplar and birch; maple, alder and oak may also contribute	• Season starts mid-to-late May, a couple of weeks later in northern areas • Highest concentration: latter part of May to mid-June	• Ragweed season in Southern Ontario and Southwestern Quebec starts from early-to-mid August • Highest concentration: late August/early September • Stops at first frost (variable) • Nettle and plantain can also contribute	• Throughout spring, summer and fall months • Concentrations may be higher late summer to fall months in Quebec • Alternaria and Cladosporium are the predominant outdoor moulds
Maritimes & Newfoundland/ Labrador	• Season: late March to last week of June • Primarily birch and poplar • Alder, maple, oak and ash may also contribute	• Season: mid-May to end of September • Highest concentration: early June	• Ragweed: early August to end of September	• Levels higher during the late summer and early fall months • Alternaria and Cladosporium are the predominant moulds

Typically, SCIT consists of two phases: a build-up phase (also known as up-dosing or induction) and a maintenance phase. During the build-up phase, the patient receives weekly injections, starting with a very low dose, with gradual increases in dose over the course of 3–6 months. The frequency of injections during this phase generally ranges from 1 to 3 times per week, although more rapid build-up schedules are sometimes used. After this period, the patient has usually built-up sufficient tolerance to the allergen such that a maintenance (therapeutic) dose has been reached. During the maintenance phase, the patient generally receives injections of the maintenance dose every 4 to 6 weeks for venom and every 4 weeks for inhalant allergens, usually for a period of 3 to 5 years. After this period, many patients experience a prolonged, protective effect and, therefore, consideration can be given to stopping therapy [9]. In the case of venom immunotherapy, a longer duration of therapy may be considered depending on patient risk factors for recurrence.

Accelerated schedules, such as rush or cluster immunotherapy, may also be used and involve the administration of several injections at increasing doses on a single visit. With cluster immunotherapy, two or three injections (at increasing doses) are given sequentially in a single day of treatment on non-consecutive days. Rush immunotherapy entails administering incremental doses of the allergen at intervals varying between 15 and 60 min over 1 to 3 days until the target maintenance dose is achieved. Although accelerated schedules offer the advantage of achieving the therapeutic dose much earlier than conventional immunotherapy schedules, they are also associated with an increased risk of systemic reactions [5, 9] and are not typically used in Canada for respiratory allergies. Although the safety profile of rush protocols for venom immunotherapy is good [10], these accelerated protocols may also be associated with an increased risk of systemic allergic reactions [49].

Pre-seasonal SCIT is a condensed treatment regimen administered a few weeks prior to the onset of the pollen season. These injections commonly incorporate an adjuvant, such as aluminum hydroxide or microcrystalline tyrosine, to amplify the antigen-specific immune response. While advantageous, this method may exhibit lower efficacy compared to conventional SCIT [50]. Furthermore, they are less easily accessed commercially in Canada.

Patients receiving maintenance immunotherapy should be followed regularly to: assess the efficacy of treatment; monitor adverse reactions; assess patient compliance with therapy; and determine whether immunotherapy can be discontinued or if dose/schedule adjustments are required. For example, dose reductions may need to be considered during periods when the patient is exposed to increased allergen levels or when he/she is experiencing an exacerbation of symptoms. Dose reductions are also necessary during interruptions in dosing schedules, and when a new vial of immunotherapy is started due to loss of extract potency over time.

At present, there are no specific tests or clinical markers that will distinguish between patients who will relapse and those who will remain in long-term clinical remission after discontinuing allergen immunotherapy. Therefore, the decision to continue immunotherapy beyond 3 to 5 years should be based on individual patient factors such as the severity of the disease, benefits sustained from treatment, reaction history, patient preference, and treatment convenience [9].

### Sublingual immunotherapy (SLIT)

SLIT offers multiple potential benefits over SCIT including the comfort of avoiding injections, the convenience of home administration, and a favourable safety profile [6]. SLIT involves placing the allergen extract, in rapidly dissolving tablet or liquid form, under the tongue until completely dissolved, and it can be self-administered following medical supervision of the first dose. It is currently available for the treatment of birch tree, grass and ragweed allergy, as well as house dust mite-induced allergic rhinitis (with or without conjunctivitis). At present, five SLIT products are available in Canada: Itulatek, Oralair, Grastek, Ragwitek and Acarizax (see Table 2) [6]. SLIT should only be administered using these Health Canada approved products.

## Safety

### Subcutaneous immunotherapy (SCIT)

SCIT is generally safe and well-tolerated when used in appropriately selected patients. However, local and systemic reactions may occur. Local reactions, such as redness or itching at the injection site, occur in approximately 35% of patients and it is uncertain whether these local reactions are associated with a higher risk of systemic reactions [6]. Local reactions can generally be managed with local treatment (e.g., cool compresses or topical corticosteroids) or oral antihistamines. Systemic reactions occur in approximately 1-12.7% of patients on inhalant SCIT [6] and can be mild to severe. The most severe reaction is anaphylaxis. Fatal anaphylactic reactions are rare; one study found no fatalities in approximately 8.1 million injections [6].

There are numerous signs and symptoms of anaphylaxis that involve the skin, gastrointestinal and respiratory tracts, and cardiovascular system (see Table 4) [51] (for a more detailed review on anaphylaxis, please see *Anaphylaxis* article in this supplement). These symptoms typically develop within 30 min after the immunotherapy injection. In studies of anaphylactic fatalities secondary to skin tests and AIT, most documented fatalities (73%) occurred within 30 min of the injection [6]. It is important to note that the signs and symptoms of anaphylaxis are unpredictable and may vary from patient to patient. Therefore, the absence of one or more of the common symptoms listed in Table 4 does not rule out anaphylaxis and should not delay immediate treatment [6]. Note that Cox et al. have proposed a modified grading system for severe allergic reactions that may allow for more consistent reporting of these reactions and better safety comparisons across different venues and treatment protocols [52].

**Table 4:** Signs and symptoms of anaphylaxis [51]

Sign/symptoms	Incidence
Urticaria, angioedema	87%
Dyspnea	59%
Dizziness, syncope	33%
Diarrhea, abdominal cramps	29%
Flushing	25%
Upper airway edema	21%
Nausea, vomiting	20%
Hypotension	15%
Rhinitis	8%
Itch without rash	5%
Seizure	1%

In the event of anaphylaxis, the treatment of choice is epinephrine administered by intramuscular injection into the lateral thigh (see *Anaphylaxis* article in this supplement for more information on the management of anaphylaxis). Recently, intramuscular epinephrine at a dose of 0.5 mg has been shown to be safe and effective for the treatment of anaphylaxis from SCIT [53].

Adjunctive therapies such as antihistamines, bronchodilators and systemic corticosteroids may also be used, but should never be given prior to or replace epinephrine in the treatment of anaphylaxis. In severe cases, intravenous saline or supplemental oxygen may be required [5, 6, 9].

Following a systemic reaction to immunotherapy, consideration should be given to reducing the therapeutic dose or to possibly discontinuing therapy, particularly if the patient has repeated systemic reactions following injections [5, 6, 9].

### Sublingual immunotherapy (SLIT)

The most common side effects of SLIT are local reactions such as oral pruritus, throat irritation, and ear pruritus [6]. These symptoms typically resolve after the first week of therapy. Pretreatment with non-sedating antihistamines may be helpful for local symptoms.

There is a very small risk of more severe systemic allergic reactions with SLIT and, therefore, some allergists may offer the patient an epinephrine autoinjector in case a reaction occurs at home. The risk of systemic allergic reactions is much lower with SLIT compared to SCIT [6]. As mentioned earlier, SLIT has been associated with new or worsening EoE [6] (see *Eosinophilic Esophagitis* article in this supplement) and, therefore, SLIT is contraindicated in patients with EoE. It is also important to note that some SLIT products contain fish-derived gelatin, and vegans or fish-allergic patients should be forewarned of this.

## Conclusions

AIT is a potentially disease-modifying therapy that is effective for the treatment of allergic rhinitis/conjunctivitis, allergic asthma and stinging insect hypersensitivity, as well as atopic dermatitis associated with HDM or aeroallergen sensitization. AIT has been associated with a shift from Th2 to Th1 immune responses, and the production of T regulatory cells that dampen the immune response to relevant allergens. When used in appropriately selected patients, AIT (particularly SLIT) is extremely safe. However, because this form of therapy is associated with a risk of anaphylactic reactions, it should only be prescribed by physicians who are adequately trained in the treatment of allergy. Furthermore, SCIT should be administered only by physicians who are equipped to manage life-threatening anaphylaxis.

## Data Availability

Not applicable.

## Declarations

### About this supplement

This article has been published as part of Allergy, Asthma & Clinical Immunology, Volume 20 Supplement 03, 2024: Practical Guide for Allergy and Immunology in Canada 2024. The full contents of the supplement are available at https://aacijournal.biomedcentral.com/articles/supplements/volume-20-supplement-3

## Abbreviations

ACE: Angiotensin-converting enzyme
AIT: Allergen immunotherapy
EoE: Eosinophilic esophagitis
FEV_1_: Forced expiratory volume in 1 s
GINA: Global Initiative for Asthma
HDM: House dust mite
ICS: Inhaled corticosteroids
IgA: Immunoglobulin A
IgE: Immunoglobulin E
IgG: Immunoglobulin G
IL-10: Interleukin 10
IL-5: Interleukin 5
SCIT: Subcutaneous immunotherapy
SLIT: Sublingual immunotherapy
SPT: Skin prick testing
TGF: Transforming growth factor
Th2: T helper cell type-2
